# From entrepreneurial passion to business model innovation: The role of entrepreneurial learning and curiosity

**DOI:** 10.3389/fpsyg.2022.1028906

**Published:** 2022-10-12

**Authors:** Shuli Zou

**Affiliations:** Department of Business English, Foreign Languages School, Huanghuai University, Zhumadian, China

**Keywords:** entrepreneurial passion, entrepreneurial learning, curiosity, business model innovation, PLS-SEM 4, China

## Abstract

The impact of entrepreneurial passion on business model innovation has become a research focus in the field of entrepreneurship. This study aimed to examine the effect of entrepreneurial passion on entrepreneurial learning and business model innovation. The study used partial least square-structural equation modeling (PLS-SEM 4) to test the hypotheses on a sample of 389 manufacturing and technology enterprises in the South region of Henan province China. The results of this study revealed that entrepreneurial passion has a positive and significant influence on entrepreneurial learning and business model innovation. The findings of this study showed that entrepreneurial learning positively and significantly mediates the relationship between entrepreneurial passion and business model innovation. Furthermore, the results of this study indicated that curiosity positively and significantly moderates the relationship between entrepreneurial passion and entrepreneurial learning. Lastly, the results are discussed with implications and limitations, which open up future research perspectives.

## Introduction

Entrepreneurship has now become a significant economic factor in this modern era. It is widely accepted that individual entrepreneurs play a lead role in starting new business ventures and even often in the early evolution of industries (Jiatong et al., 2021). Entrepreneurship is considered a “major catalyst” in the economic progress of developing nations (Morris et al., 2013; Syed et al., 2020). Entrepreneurial passion is defined as entrepreneurs’ intense and conscious feelings regarding various entrepreneurial activities and roles. Entrepreneurial passion is one of the most discussed areas in the entrepreneurship process, and it helps make entrepreneurs more novice and successful. Besides, entrepreneurial passion is a distinct feature that separates entrepreneurs from normal individuals having no skills (Cardon and Kirk, 2015; Biraglia and Kadile, 2017). If entrepreneurs have enough passion and learning, they can successfully make the business model innovation smooth. When entrepreneurs are passionate, this factor increases the effectiveness of business model innovations (Martins et al., 2015).

Entrepreneurial passion helps entrepreneurs discover potential opportunities through their enthusiasm and passion. Still, they are unfamiliar with converting these opportunities into reality for value creation. It indicates that all entrepreneurs are not passionate about accomplishing the goal of starting their business models (Fesharaki, 2019; Snihur and Zott, 2020). Entrepreneurial passion contributes to developing new businesses during the growth phases of companies (Baron and Hmieleski, 2018; Khan et al., 2022). Several business startups have been started in China due to the growing importance and awareness regarding innovation and entrepreneurship. Most startups are associated with the manufacturing and e-commerce sectors. Entrepreneurs can easily start their businesses in these sectors, but it is not easy to grow and survive such companies in the market. There are multiple reasons for this difficulty, such as lack of legitimacy, lack of resources, facing slack phases, and need for newness (Davis et al., 2017).

Prior researchers argued that business model innovation has become a significant practice for business startups to develop and sustain in the competitive market in the long run. Numerous new business ventures have achieved success by focusing on innovative business model (Spieth et al., 2014; Foss and Saebi, 2017). The instances of business model innovation include Alipay and WeChat, which have succeeded in achieving success because of their competitive and innovative business models. However, until now the knowledge is scarce regarding the emerging trend of business model innovation and research gap on investigating the phenomenon of business model innovation is still under-explored (Euchner and Ganguly, 2014; Snihur and Zott, 2020).

Ma et al. (2017) said that when entrepreneurs are passionate, they search for new opportunities and knowledge which help them accomplish their long-term goals. Entrepreneurial passion involves such abilities and skills in gathering information and scanning new opportunities. It allows entrepreneurs recognize the approaches leading toward business model innovation. Therefore, entrepreneurial passion is one of the key factors influencing business model innovation (Zhao et al., 2021). Entrepreneurial passion helps entrepreneurs discover potential opportunities through their enthusiasm and passion. Still, they are unfamiliar with converting these potential opportunities into reality for value creation (Santos and Cardon, 2019). It indicates that all entrepreneurs are not passionate about accomplishing the goal of starting their business models.

This research analyzes the framework of business model innovation and entrepreneurial passion. The intention model proposes that perceptions derive actions from feasible and desirable opportunities (Feng and Chen, 2020; Zhao et al., 2021). It indicates that when entrepreneurs investigate feasible and desirable opportunities, they can achieve the goal of business model innovation. Entrepreneurs can make their choices feasible and desirable when they have entrepreneurial learning. Entrepreneurial learning plays a significant role in making choices and opportunities feasible according to the resource-based view (Boldureanu et al., 2020). Entrepreneurial learning helps increase the entrepreneurs’ perceptions that their actions and choices are feasible by enhancing the knowledge base (Wang and Chugh, 2014). Therefore, entrepreneurs are better positioned to take action on business startups.

Curiosity is considered one of the most influential factors affecting the behavior of entrepreneurs (Kashdan and Silvia, 2009; Arikan et al., 2020). When entrepreneurs have curiosity, they have the intense desire to identify uncertain and challenging methods and opportunities. They try to learn new techniques to help them start business startups. Entrepreneurial passion has a favorable effect on learning when combined with curiosity. Curiosity remains under-investigated in the field of entrepreneurship; thus, this study fulfills this research gap and investigates the following research questions;

RQ1: What is the impact of entrepreneurial passion on business model innovation?
RQ2: Does entrepreneurial learning mediates the relationship between entrepreneurial passion and business model innovation?
RQ3: Does curiosity moderates the relationship between entrepreneurial passion and entrepreneurial learning?

## Theory and literature review

### Business model innovation

Business model uses evidence, data, and logic to increase the customer value proposition (Massa et al., 2017). It establishes a structure of costs and revenues for companies that deliver high customer value (Teece, 2010). Morris et al. (2013) argued that it explains why companies generate income. The business model comprises three factors: governance, structure, and content. According to Snihur and Zott (2020), governance includes governing activities and transactions based on control issues. The structure indicates the linkage of activities, and the content includes various system activities. The core of business model innovation, thus, entails regulating transactions, connecting activities in novel ways, and adding additional activities through new partners or in exciting ways.

Business model innovation has become a prominent topic for debate in this age (Trimi and Berbegal-Mirabent, 2012). It helps entrepreneurs enhance their chances of success and indicates value creation. Martins et al. (2015) investigated that how entrepreneurs succeed in business model innovation. However, limited studies have examined the factors influencing business model innovation. Entrepreneurs have thinking patterns and creativity skills that help them to start a new business. Furthermore, the literature on business model innovation in the context of quantitative investigation is empirically less studies by the prior researchers (Amit and Zott, 2015).

### Intentions model

Krueger et al. (2000) presented the intentions model to analyze how entrepreneurs can succeed in business model innovation. Shepherd and Krueger (2002), provided a framework for comprehending entrepreneurial activities and examine entrepreneurs’ cognitive behavior. Krueger (2017) noted that the intentions model is based on entrepreneurs’ perceptions of feasibility and desirability impacting their actions and intentions. The desirability of entrepreneurs can be considered the utility desired from entrepreneurship results. Feasibility is associated with the beliefs of entrepreneurs that they can desirably complete their responsibilities.

The existing literature indicates that the cognition of entrepreneurs impacts entrepreneurial actions and intentions (Douglas, 2017; Cai et al., 2021). Entrepreneurial passion is a significant factor in the cognition of entrepreneurs that impacts entrepreneurial activities. Entrepreneurial passion is the ability of entrepreneurs to analyze various potential opportunities which others have ignored. According to Puhakka (2011), entrepreneurial passion can explore new solutions for solving business problems and tapping new business opportunities. Ma and Huang (2016) have focused on the factors of entrepreneurial passion. Still, a few researchers have examined how entrepreneurial passion can impact the outcomes of business startups.

Moreover, entrepreneurial passion helps entrepreneurs identify the market cues to tap into more opportunities. Entrepreneurs will achieve their long-term objectives and value creation if they take advantage of these opportunities (Anjum et al., 2021). Entrepreneurs evaluate and consider entrepreneurial activity feasible and achievable depending on the various opportunities recognized by entrepreneurial passion. Entrepreneurs will carry out this activity if they believe it is practicable (Zhao et al., 2021).

### Knowledge-based view

The main contribution of knowledge in identifying significant opportunities is highlighted by the knowledge-based view (KBV). According to Spender (1996), creating value and gaining a competitive advantage with knowledge is possible. According to this theory, innovation combines knowledge to create new developments (Foss et al., 2013). Zhou and Li (2012) argued that the company’s existing knowledge is limited in its capacity and scope to understand and apply new knowledge to innovativeness. Henceforth, knowledge acquisition is essential to achieve innovation. Bierly and Daly (2007) noted that business startups employ a method to learn through entrepreneurial learning. It is a practical strategy for gaining a competitive edge. Developing entrepreneurial knowledge is a process people go through in their professional careers to increase their effectiveness and managerial capabilities for newly established enterprises. Entrepreneurs combine and update their knowledge through learning. The basics of entrepreneurial learning are what entrepreneurs do while identifying new opportunities in establishing or managing existing businesses (Wang and Chugh, 2014).

### Hypotheses development

#### Entrepreneurial passion and business model innovation

Entrepreneurial passion helps entrepreneurs identify, analyze, and evaluate market information (Kiani et al., 2020). Entrepreneurial passion helps entrepreneurs and managers in multiple ways. First, it helps discover and fulfill the customer’s requirements by discovering the information in the market, which is the core activity in accomplishing business model innovation (Murnieks et al., 2020). Entrepreneurs face difficulties in analyzing and satisfying the needs of customers in a better way. Zott and Amit (2007) noted that entrepreneurial passion increases the willingness of entrepreneurs to increase value by fulfilling the customers’ demands. Entrepreneurs should be engaged in new approaches and activities, governing tasks, and associating activities to lead toward business model innovation.

Second, entrepreneurial spirit permits entrepreneurs to examine new market activities by inventively fusing various pieces of knowledge. It can help entrepreneurs innovate their business models (Amato et al., 2017). Tang et al. (2012) argued that association combines different information and coherently assembles them. Entrepreneurs find new markets and ways of business startups based on associating unconnected information. The internet’s improvements, the internet drop in communication costs, and newly developing initiatives on the internet have all made it possible for new concepts and methods of value creation to emerge. Passionate business owners integrate information technologies into their operations and create exchange mechanisms that foster the development of novel business models (Zott et al., 2011).

Third, entrepreneurial passion supports entrepreneurs to analyze the external constraints of the environment, which are a big challenge for innovation (Collewaert et al., 2016). A business model’s effectiveness is determined by its integration of industry, technology, and legal norms. The external forces influence the creative alternatives, feasibility, and desirability of business model innovation. The execution of business model operations is also impacted. Entrepreneurs with much passion can examine these boundaries and combine the research done before with the association (Hargadon and Douglas, 2001). They discover that boundaries can provide entrepreneurs with better opportunities to adopt new transaction forms. Therefore, environmental limits can be seen as a significant obstacle and support in creating business models by entrepreneurs (De Mol et al., 2020). Consequently, the following hypothesis is proposed in light of the discussion:

**H1:** Entrepreneurial passion has a positive influence on business model innovation.

#### Entrepreneurial passion and entrepreneurial learning

The experiential learning theory was used to examine the learning role in entrepreneurship. Corbett (2005) said that exploitation and identification of opportunities affect learning processes. Jabarullah and Hussain (2019) argued that entrepreneurs are encouraged to discover opportunities and develop passion through learning the process of entrepreneurship. According to Bonneville-Roussy et al. (2013), entrepreneurial learning and passion positively correlate, when entrepreneurs are more passionate, they are engaged in the effective learning process, which boosts entrepreneurship. Nobanee and Dilshad (2020) observed that the learning outcomes of entrepreneurs are concrete and emotional. Some psychological traits impact individuals’ entrepreneurial intentions, but emotional characteristics indicate changes through the experiential learning process. Somjai and Sangperm (2019) noted that emotion is one of the most significant learning elements; thus, learning and emotion boost entrepreneurship. Entrepreneurship should be considered an emotional process that combines high commitment, lack of control, uncertainty, excitement, and fear.

Prior researchers neglected the cognitive element (Grégoire et al., 2011; Lackéus, 2012). This idea has been used to convey various meanings, including love and cognition, as well as emotions. The terms fun, excitement, and passion indicate emotions that help establish a new firms. The quality and amount of emotional involvement are the reason for the development and survival of the venture. It takes emotional perspectives to develop new ways to look at entrepreneurship. The creation of a setting conducive to successful learning requires emotional exposure (Gondim and Mutti, 2011). Therefore, entrepreneurial passion helps entrepreneurs engage in entrepreneurial learning. Based on the “self-determination theory,” the impact of entrepreneurial passion on entrepreneurial learning are positively correlated (Sriyakul and Jermsittiparsert, 2019). In light of the discussion that was just conducted, it is therefore suggested that:

**H2:** Entrepreneurial passion is positively related to entrepreneurial learning.

#### The mediating effect of entrepreneurial learning

Entrepreneurial passion assists entrepreneurs in exploring several approaches toward entrepreneurs’ success in business model innovation. Researchers contend that looking into the association between business model innovation and entrepreneurial passion is imperative (Krueger, 2007; Kiani et al., 2021). The intentions model provides the foundation for evidence of this relationship. According to the intentions model, entrepreneurs’ conceptions of achievable and desirable results motivate their actions and intentions (Franzoni and Tenca, 2021). The decision to take a particular action depends on how desirable the opportunity is to oneself. Entrepreneurs who want to proceed in action wait for feasibility before forming entrepreneurial intentions. Entrepreneurs can create new business models by combining desirability and feasibility (Douglas, 2017). Entrepreneurial passion offers various methods for achieving desired business model innovation. However, business owners should take the necessary steps to view these strategies as actionable. As a result, the technique by which business owners assess the feasibility of many alternative strategies acts as a mediator between entrepreneurship passion and business model innovation.

According to Keh et al. (2002), the knowledge-based view supports the idea that entrepreneurial learning is a fundamental mediator. Entrepreneurs should gain the necessary skills to influence future circumstances to increase their feasibility. Entrepreneurial learning helps business owners in acquiring the necessary knowledge and strengthens their ability to act. Knowledge based view KBV focuses that the main functions of an organization include exploring, integrating, and applying the knowledge (Grant, 1996; Sousa and Hendriks, 2006). The knowledge base organizations can foster its innovativeness and help entrepreneurs control future situations. Through entrepreneurial learning, entrepreneurs get additional information that may be translated into the capacity to influence situations in the future. By gaining and transforming knowledge into their capabilities, they discover activities, governance, and connecting structures. All these elements encourage business model innovation (Duarte Alonso et al., 2022). Experiential learning provides the skills and information needed to implement strategies, while entrepreneurial passion provides a variety of methods for achieving business model innovation (Zhao et al., 2021). Entrepreneurs will achieve business model innovation after combining these two factors. Therefore, entrepreneurial learning mediates entrepreneurial passion and business model innovation. As a result, the following hypotheses are formulated:

**H3: E**ntrepreneurial learning has a positive impact on business model innovation.

**H4:** Entrepreneurial learning mediates the association between entrepreneurial passion and business model innovation.

#### The moderating role of curiosity

One of the key factors influencing people’s behavior is thought to be curiosity. Curiosity can be explained as a desire to analyze and identify risky, challenging, and new events (Kashdan and Silvia, 2009; Syed et al., 2020). Entrepreneurs are passionate about learning new ideas and learning novel opportunities. As a result, when entrepreneurs are curious, the relationship between their curiosity and their learning is strengthened (Jeraj and Antoncic, 2013). According to self-regulatory theory, curiosity can help entrepreneurs concentrate on their goals and gather information about their industry (Tolentino et al., 2014). Curiosity increases innovativeness among entrepreneurs because it needs specific information and integration across various disciplines and fields. The self-regulatory theory proposes the positive impact of curiosity. Several researchers have investigated that the curiosity of entrepreneurs is associated with entrepreneurial passion and entrepreneurial learning (Celik et al., 2016; Peljko et al., 2016).

Kashdan et al. (2018) argued that curious entrepreneurs can handle even the most difficult challenges to achieve their objectives. The self-regulatory theory proposes that curiosity provides action orientation, energy, and persistence toward specific goals. Hence, curiosity increases entrepreneurs’ interest and attention toward their specific objectives (Celik et al., 2016). Therefore, it is observed that the favorable effects of curiosity improve the association between entrepreneurial learning and passion (Liu et al., 2017). Thus, the following hypotheses are framed:

**H5:** Curiosity has a positive effect on entrepreneurial learning.

**H5a:** The relationship between entrepreneurial passion and entrepreneurial learning is moderated by curiosity.

#### Conceptual model

**FIGURE 1:**
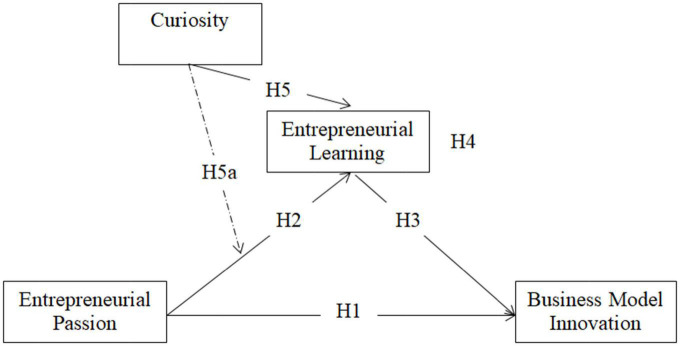
Conceptual model.

This study examines the role of entrepreneurial passion on business model innovation through entrepreneurial learning and curiosity based on a knowledge-based view and the intentions model. Figure 1 indicates the conceptual model of this research.

## Materials and methods

### Population and sample

The nature of this study was cross-sectional, and data was collected from the manufacturing and services enterprises in the South region of Henan province, China. The economy of Henan province is much developed, and many industries are situated that contribute to China’s national economy. Recently, the Chinese government has implemented a new strategy to facilitate young entrepreneurs for starting new business ventures and startup activities. Therefore, many new ventures have merged in this region, providing a perfect context for conducting a survey study on entrepreneurial passion, entrepreneurial learning, and business model innovation.

Moreover, we used a convenience sampling technique and selected 500 small and medium enterprises SMEs in Henan province, China. We visited manufacturing and technology enterprises one by one and surveyed through face-to-face meetings with CEO’s and founders who were easily available to meet with us. Most participants agreed to participate in this study survey, and some were busy in official meetings. Finally, we received 389 completed surveys, with a participation rate of 77.8%. Furthermore, to ensure the acceptability of the sample, we performed a *t*-test to find the non-response bias in the collected data. The results of the *t*-test for gender, entrepreneur age, education, and enterprise age were insignificant, and there was less possibility of non-response bias. The original draft of the survey was in English and then translated into Chinese. In order to collect more reliable data, a standard back-to-back translation process was done following the procedures of Schaffer and Riordan (2003). According to the prior studies, the back-translation method confirms the translation accuracy and ensures that all the measurement constructs can be used in the Chinese context (Tsui et al., 2007; Liao et al., 2010).

Moreover, to reduce common method variance bias, we used two approaches in this study, i.e., procedural and statistical. First, we split independent (Time 1) and dependent variables (Time 2) in the data collection and also provided the guideline to respondents to generate the code (i.e., gender, age, and any key identifications) to match the responses at Time 2 (Conway and Lance, 2010). Second, we used Harman’s one-factor test to identify the common method variance bias (Podsakoff et al., 2003). The findings showed that the total variance of the first factor is 31.42%, well above the threshold value of 50%, suggesting that there is no issue of common method variance (Chang et al., 2020).

### Demographical profile

Table 1 shows the demographical and descriptive statistics of the sample. Among the valid responses, (63.5%) were filled by males and (36.5%) by females. The age range was from 21 to 30 (12.3%); 31 to 40 (31.1%); 41 to 50 (49.6%), and 51 to 60 (6.9%). The education rate of the respondents was (11.3%) school; (23.7%) college or technical education; (49.9%) bachelor and (15.2%) had a master’s or higher degree. Most enterprises were working for more than (27.2%) years, and (72.8%) were newly established.

**TABLE 1:** Demographical profile.

Demographics	Frequency	Percent	Mean	SD	Skewness	Kurtosis
**Gender**			1.37	0.482	0.563	–1.692
Male	247	63.5%				
Female	142	36.5%				
**Age**			2.51	0.798	–0.359	–0.429
21–30 years	38	12.3%				
31–40 years	121	31.1%				
41–50 years	193	49.6%				
51–60 years	27	6.9%				
**Education**			2.69	0.864	–0.415	–0.412
School	44	11.3%				
College and technical	92	23.7%				
Education	194	49.9%				
Bachelors	59	15.2%				
Masters or above degree						
**Enterprise age**			1.73	0.446	–1.026	–0.952
1–3 years	106	27.2%				
6–15 years	283	72.8%				
Total	389	100%				

### Measures

All the measurement items were adopted from the prior researcher’s work on entrepreneurial passion, curiosity, entrepreneurial learning, and business model innovation. We used 5-point Likert scales ranging from 1-strongly disagree to 5-strongly agree (see Appendix).

#### Entrepreneurial passion

To measure entrepreneurial passion, we used five measurement items adopted from the study (Li et al., 2020). This scale was recommended by prior researchers (Cardon et al., 2013, 2017). A sample item, “searching for new ideas for products/services to offer is enjoyable to our enterprise.” (The Cronbach’s alpha for entrepreneurial passion was = 0.947).

#### Curiosity

To assess the moderating role of curiosity in the relationship between entrepreneurial passion and entrepreneurial learning, we adopted six items scale associated with curiosity and exploration inventory-II, developed by Kashdan et al. (2009). Previous researchers have already used this scale (Syed et al., 2020). The two dimensions of curiosity- stretching (seeking out new knowledge and new experiences) and embracing (willingness to embrace uncertainty in everyday life). (The Cronbach’s alpha for curiosity was = 0.938).

#### Entrepreneurial learning

To measure entrepreneurial learning, we used five measurement constructs from the study (Atuahene-Gima and Murray, 2007). This scale was tested by prior researchers (Zhao et al., 2021). A sample item “collected novel information and ideas that went beyond our current market and technologies experiences.” (The Cronbach’s alpha for entrepreneurial learning was = 0.941).

#### Business model innovation

Business model innovation was measured using eight items scale from the prior study by Zott and Amit (2007) and Zhao et al. (2021). A sample item is “the business model creates a new profitable way.” (The Cronbach’s alpha for business model innovation was = 0.956).

## Results

### Data analysis method

This study applied partial least squares structural equation modeling PLS-SEM approach to verify the proposed hypotheses. According to previous researchers, PLS excludes strict assumptions that underlie the utmost possibility method and protects against misleading alternatives and factor uncertainty (Hair et al., 2012, 2013). Furthermore, unlike covariance-based SEM techniques, PLS-SEM is unlikely to be affected by sample size limitations and can be used for larger sample sizes (Hair et al., 2019).

### Measurement model

**FIGURE 2:**
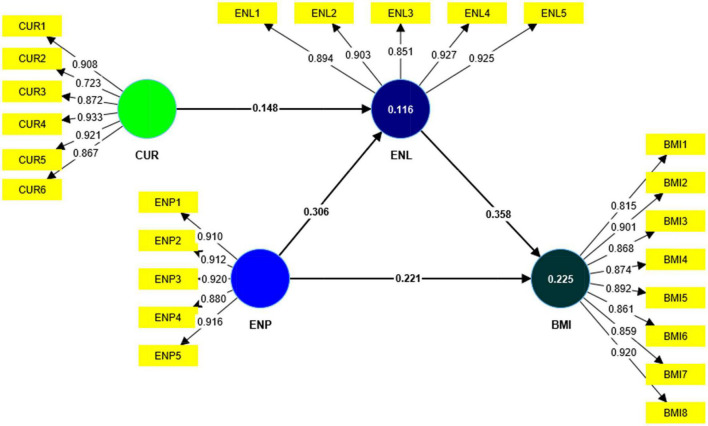
Measurement model.

The measurement model was assessed through factor loadings, Cronbach’s alpha (CA), composite reliability (CR), and average variance extracted (AVE). Table 2 and Figure 2 show that standardized factor loadings of the indicators on their particular construct was more significant than 0.70, and all the values of factor loadings met the criteria proposed by Henseler et al. (2009) and Hair et al (2020).

**TABLE 2:** Factor loadings.

Indicators	BMI	CUR	ENL	ENP
BMI1	0.815	0.000	0.000	0.000
BMI2	0.901	0.000	0.000	0.000
BMI3	0.868	0.000	0.000	0.000
BMI4	0.874	0.000	0.000	0.000
BMI5	0.892	0.000	0.000	0.000
BMI6	0.861	0.000	0.000	0.000
BMI7	0.859	0.000	0.000	0.000
BMI8	0.920	0.000	0.000	0.000
CUR1	0.000	0.908	0.000	0.000
CUR2	0.000	0.723	0.000	0.000
CUR3	0.000	0.872	0.000	0.000
CUR4	0.000	0.933	0.000	0.000
CUR5	0.000	0.921	0.000	0.000
CUR6	0.000	0.867	0.000	0.000
ENL1	0.000	0.000	0.894	0.000
ENL2	0.000	0.000	0.903	0.000
ENL3	0.000	0.000	0.851	0.000
ENL4	0.000	0.000	0.927	0.000
ENL5	0.000	0.000	0.925	0.000
ENP1	0.000	0.000	0.000	0.910
ENP2	0.000	0.000	0.000	0.912
ENP3	0.000	0.000	0.000	0.920
ENP4	0.000	0.000	0.000	0.880
ENP5	0.000	0.000	0.000	0.916

ENP, Entrepreneurial passion; ENL, Entrepreneurial learning; CUR, Curiosity; BMI, Business model innovation.

Moreover, Table 3 results illustrate that all factors had a satisfactory composite reliability CR greater than 0.80; Cronbach’s alpha values were more significant than 0.70, and AVE values were greater than 0.50, as suggested by Sarstedt et al. (2014). Furthermore, we followed Fornell and Larcker (1981) and Hetrotrait-Monotrait HTMT ratio criteria to test the discriminant validity. According to Fornell and Larcker (1981), the criterion for measuring the discriminant validity is a square root of the AVE, and the values of correlations were below the result of discriminant validity (Fornell and Larcker, 1981; Henseler et al., 2015). As per the criteria of the HTMT ratio, the values should be less than 0.85 (Roemer et al., 2021). Therefore, it is observed that the highest attained HTMT value was (0.441), which was below the suggested value of 0.85. Thus, Tables 4, 5 results show that all the constructs fully meet the criteria for discriminant validity.

**TABLE 3:** Construct reliability and validity.

Constructs	CA	CR	AVE	VIF
BMI	0.956	0.963	0.764	–
CUR	0.938	0.950	0.763	1.000
ENL	0.941	0.955	0.811	1.104
ENP	0.947	0.959	0.824	1.104

ENP, Entrepreneurial passion; ENL, Entrepreneurial learning; CUR, Curiosity, BMI, Business model innovation; CA, Cronbach’s alpha; CR, Composite reliability; AVE, Average variance extracted; VIF, Variance inflation factor.

**TABLE 4:** Discriminant validity (Fornell-Larcker criterion).

Constructs	BMI	CUR	ENL	ENP
BMI	0.874			
CUR	0.053	0.874		
ENL	0.426	0.149	0.900	
ENP	0.330	0.002	0.306	0.908

ENP, Entrepreneurial passion; ENL, Entrepreneurial learning; CUR, Curiosity; BMI, Business model innovation.

**TABLE 5:** Discriminant validity (Heterotrait-Monotrait ratio HTMT).

Constructs	BMI	CUR	ENL	ENP
BMI	0.000	0.000	0.000	0.000
CUR	0.079	0.000	0.000	0.000
ENL	0.441	0.146	0.000	0.000
ENP	0.340	0.033	0.324	0.000

ENP, Entrepreneurial passion; ENL, Entrepreneurial learning; CUR, Curiosity; BMI, Business model innovation.

### Structural model

Various quality scores like the coefficient of determination (R^2^) and SRMR were used to analyze the structural model results. Table 6 shows the R^2^ and SRMR values. R^2^ values for endogenous constructs were used to evaluate model fit and determine how well data points matched a line or curve. According to Chin (1998), R^2^ levels can be categorized as small (0.02 to R^2^ 0.13), medium (0.13 to R^2^ 0.26), or large (R^2^ 0.26), depending on the R^2^ level. The endogenous constructs’ R^2^ statistic values were utilized to test model fit (Henseler et al., 2015). The values of entrepreneurial learning were (*R*^2^ = 0.116), and business model innovation was (R^2^ = 0.225), which indicates a medium effect size. Furthermore, SRMR (standardized root mean squared residual) ought to be equivalent to or even less than 0.08 (Hair et al., 2013), and our model’s SRMR is (0.047), which fulfills this set of criteria.

**TABLE 6:** R^2^ and model fitness.

Constructs	*R* ^2^	*R*^2^ adjusted	SRMR value
BMI	0.225	0.221	0.047
ENL	0.116	0.111	

ENL, Entrepreneurial learning, BMI, Business model innovation; SRMR, Standardized root mean squared residual.

### Hypotheses testing

**FIGURE 3:**
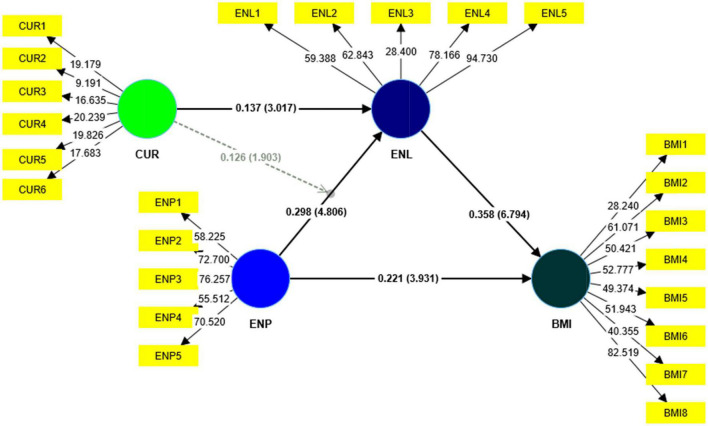
Structural model.

The hypotheses were analyzed with the Smart-PLS software version 4 using the 5,000 bootstrapping method. Table 7 and Figure 3 show the outcomes. All the findings were statistically significant. The results of the H1 show that entrepreneurial passion has a significant effect on business model innovation (β = 0.221; *t* = 3.931; *p* < 0.000). As a result, H1 was accepted. Moreover, in the meantime, H2 findings indicate that entrepreneurial passion has a significant influence on entrepreneurial learning (β = 0.298; *t* = 4.806; *p* < 0.000). As a result, H2 was accepted. Furthermore, H3 results show that entrepreneurial learning positively and significantly impacts business model innovation (β = 0.358; *t* = 6.794; *p* < 0.000). Thus, H3 was accepted. Additionally, H4 findings show that entrepreneurial learning positively mediates the relationship between entrepreneurial passion and business model innovation (β = 0.107; *t* = 4.196; *p* < 0.000). As a result, H4 was approved.

**TABLE 7:** Hypotheses testing.

Hypothesis	Constructs	β	*t*	*p*
H1	ENP – > BMI	0.221	3.931	0.000
H2	ENP – > ENL	0.298	4.806	0.000
H3	ENL – > BMI	0.358	6.794	0.000
**Indirect effect**				
H4	ENP – > ENL – > BMI	0.107	4.196	0.000
**Moderating effect**				
H5	CUR – > ENL	0.137	3.017	0.003
H5a	CUR – ENP – > ENL	0.126	1.903	0.040

ENP, Entrepreneurial passion; ENL, Entrepreneurial learning; CUR, Curiosity; BMI, Business model innovation.

Besides, to assess the partial or full mediation effect, the indirect effect was analyzed through variance accounted for (VAF), which indicates the ratio of the indirect effect to the total effect. According to Hair et al. (2012), if the value of VAF is larger than 0.20 and smaller than 0.80, it represents partial mediation, and if the value of VAF is greater than 0.80, it represents full mediation. Table 8 results indicate that the values of VAF are within the threshold value of 0.20–0.80% that is (32.62%). Consequently, we confirm the partial mediation. Lastly, H5 and H5a results illustrate that curiosity has a significant effect on entrepreneurial learning (β = 0.358; *t* = 6.794; *p* < 0.000) as well as positively moderated the relationship between entrepreneurial passion and entrepreneurial learning (β = 0.126; *t* = 1.903; *p* < 0.040), Hence, H5 and H5a were also supported.

**TABLE 8:** Mediation analysis.

Exogenous variable	Direct effect	Indirect effect	Total effect	VAF	Mediation	Endogenous variable
ENP	0.221	0.107	0.328	32.62%	Partial Mediation	ENL

ENP, Entrepreneurial passion; ENL, Entrepreneurial learning.

## Discussion

The current study aims to assess the influence of entrepreneurial passion on business model innovation through the mediating role of entrepreneurial learning and the moderating role of curiosity based on the knowledge-based view and the intentions model. All of the study hypotheses were confirmed. H1 contends that entrepreneurial passion is significantly impacted on business model innovation. Entrepreneurs passionate about their businesses can examine new ways to innovate and market prospects (Zott and Amit, 2007). Customers can be satisfied by passionate business owners, which is a dominant prerequisite for business model innovation. Entrepreneurs who are passionate about their businesses increase consumer value, which leads to more business model innovation. Moreover, Kiani et al. (2021), also confirm that entrepreneurs can assemble unconnected information through their passion which helps them to introduce new ways of advancement in business models. Amit and Zott (2012) argued that passionate entrepreneurs focus on reducing communication costs and emerging startups which helps them achieve the goal of innovating business models. Therefore, the previous studies are also aligned with the results of the current study.

As per H2 and H3, entrepreneurial learning is highly impacted by entrepreneurial passion and business model innovation. This finding is in line with other research studies. Jabarullah and Hussain (2019) said that entrepreneurial passion helps entrepreneurs identify new opportunities and learn more knowledge which boosts entrepreneurship. Similar results were made by Bonneville-Roussy et al. (2013), who discovered that entrepreneurial passion and learning are positively correlated. The passion among entrepreneurs enables them to participate in various learning methods, ultimately leading to organization success. Entrepreneurial learning is an important cognitive aspect that previous researchers neglected. However, entrepreneurial learning boosts the emotions of entrepreneurs, such as excitement and fun. If entrepreneurs are passionate enough, they focus on improving their learning abilities to foster innovation in their organizations. Entrepreneurial passion is a factor that can encourage involvement in efficient learning methods and processes (Sriyakul and Jermsittiparsert, 2019). The self-determination theory supports this positive relationship. Therefore, the past studies are aligned with the results of this study (Zhao et al., 2021).

H4 contends that entrepreneurial learning is a mediating factor in the association between entrepreneurial passion and business model innovation. This finding is consistent with past researchers who found this relationship significant and positive. According to Krueger (2007), entrepreneurial passion investigates many successful business model innovation strategies. Through a mediator, which in this case is entrepreneurial learning, the intentions model supports the positive relationship between business model innovation and entrepreneurial passion. According to this theory, the intentions and actions should be feasible and desirable, and business model innovation can be accomplished by combining feasibility and desirability. Nevertheless, at the same time, entrepreneurs should ensure that they implement feasible actions. From this point forward, the mediator between entrepreneurial passion and business model innovation helps business owners and entrepreneurs evaluate the feasibility of alternative entrepreneurship techniques. Besides, the knowledge-based view also supports the mediating effect of entrepreneurial learning. Entrepreneurs can develop their abilities to check the feasibility of various functions, leading to positive business model innovation. Thus, the past studies are aligned with the outcomes of this research.

According to H5 and H5a, the association between entrepreneurial learning and passion is moderated by curiosity. This finding is in line with earlier research that discovered the moderating effect of curiosity (Syed et al., 2020). According to Celik et al. (2016), entrepreneurs are passionate about discovering new, creative chances. However, the link between entrepreneurial learning and passion is strengthened when people are curious. According to the self-regulatory theory, curious entrepreneurs can better gather pertinent information for the success of organizations. When entrepreneurs learn about their fields, their curiosity leads to increased entrepreneurial learning. Similarly, Kashdan et al. (2018) said that curiosity enables entrepreneurs to accept and perform the most difficult tasks in the entrepreneurship field, ultimately strengthening their passion and learning.

### Theoretical implications

The current study highlights how entrepreneurs succeed in achieving business model innovation, but some other entrepreneurs face difficulties innovating their business models. This study makes three key theoretical advancements. It starts by looking into the significant influence of entrepreneurial passion on business model innovation. The study’s findings support the entrepreneurship discussion’s focus on business model innovation and entrepreneurial passion. According to Obschonka et al. (2017) and Zhao et al. (2021), the antecedents of business model innovation were the subject of earlier studies. This study advances the field by focusing on the influence of entrepreneurial passion on business model innovation. The role of entrepreneurial passion has not received much attention in previous studies; as a result, this study makes an important addition. This study builds on earlier studies by investigating the aspects of business model innovation.

The second part of this study examines the mediating function of entrepreneurial learning based on the knowledge-based view and the intentions model (Foss and Saebi, 2018; Snihur and Zott, 2020). The study’s findings add to the knowledge about the intentions model and connect it to the knowledge-based perspective on entrepreneurship. Entrepreneurial passion is a sign of a person’s excitement for starting a business, but a method is needed to influence the development of new business models. This study thus contributes to the body of knowledge concerning how entrepreneurs may achieve business model innovation through entrepreneurial learning (Sosna et al., 2010; Foss and Saebi, 2017). This study demonstrates how innovation requires a variety of mechanisms, from methods to the development of business model innovation. The literature on the steps of innovation realization, such as idea generation and execution, is expanded by this study. The existing literature looks at how some entrepreneurs can start their business models while others are unsuccessful. The mediating role of entrepreneurial learning, added by this study, is based on the “intentions model and a knowledge-based view.” By tying together two entrepreneurship ideas, this work significantly contributes to the field.

Third, this study reveals how curiosity moderates the relationship between entrepreneurial learning and passion. A few studies have looked at how curiosity influences entrepreneurship (Syed et al., 2020). Entrepreneurs who are more curious about starting their businesses strive to learn more and are more passionate about it. As a result, curiosity increases the association between entrepreneurial learning and passion. The results of this study suggest that additional research on curiosity in the area of entrepreneurship is necessary.

### Practical implications

This study offers numerous management implications by emphasizing how managers and entrepreneurs create business model innovation. First, this study suggests that entrepreneurs emphasize various elements while achieving business model innovation in their startups. They should be more passionate about converting their initial ideas into reality. Besides, entrepreneurs should focus on their competencies and abilities that will help them to convert their innovative ideas into reality. It includes gaining knowledge through learning and strengthening their skills. Entrepreneurs should be curious while gaining knowledge through learning because curiosity will increase their passion for learning entrepreneurial skills and ideas.

Furthermore, entrepreneurs should analyze their environment and be passionate about effectively implementing the business model innovation. If they adopt the effective entrepreneurial learning style, they will achieve their goal of business model innovation. Effective entrepreneurial learning will ensure the survival and development of their startups. This study suggests that entrepreneurial passion is necessary for success in business startups; thus, entrepreneurs should be motivated to become passionate and innovative by gaining knowledge from entrepreneurial learning. Arranging workshops and seminars can help provide a practical learning experience to entrepreneurs so they can efficiently innovate their business models.

### Limitations and future research directions

Despite the study’s significant contributions, there are several limitations. First, this research employed the research design of a cross-sectional study which restricts the investigation of the causal associations among variables. So, researchers can assess the influence of entrepreneurial passion on business model innovation through a longitudinal study based on secondary data in the future. Researchers can gather information regarding the cognitive behaviors of entrepreneurs and contact these businesses to gather data about their development and growth after some time. Also, researchers in the future can collect secondary data about the development and growth of business startups from the internet or database (Sekaran and Bougie, 2016). Second, the current study gathered data from CEOs and founders of enterprises in China, which restricts the generalizability of the research. Researchers in the future can collect data from other sectors in China to increase the generalizability of research. Data can be collected from countries to check the generalizability of research findings. Third, this research study collected data from the existing businesses; thus, there can be a bias toward survivorship. Researchers in the future can collect data from failed SMEs to generalize the results and make them more valid and reliable.

Based on the findings, this study also offers some directions for subsequent investigations. This quantitative research first examines the association between entrepreneurship passion and business model innovation. Future studies can study the phenomenon utilizing a qualitative or mixed method. In this approach, researchers can get more valuable data. They can conduct case studies or interviews to investigate the causes of business model innovation (Martins et al., 2015; Malmström and Johansson, 2017). Second, this study has concentrated on a few aspects of business model innovation. Future studies, however, might examine some new aspects of business model innovation. Third, this study does not cover four crucial business model innovation concepts. According to Osiyevskyy and Dewald (2015), future studies can examine the relationships between multiple business model innovation themes and antecedents.

## Conclusion

The aim of this study was to examine the impact of entrepreneurial passion on business model innovation with the mediating role of entrepreneurial learning and moderating effect of curiosity. This study found that entrepreneurial passion is a vital determinant of business model innovation and adds the body of knowledge on entrepreneurship. Based on a moderated mediated model, this study showed that entrepreneurial learning and curiosity influence the development of new business model innovation in the context of manufacturing and technology industries of China. Moreover, this study also investigated the effect of entrepreneurial learning and curiosity to uncover the relationship between entrepreneurial passion and business model innovation. As a result, this study contributes significant empirical evidence for the “intentions model.” It links the intentions model and the “knowledge-based view” in theoretical and conceptual considerations of entrepreneurship.

## Data availability statement

The original contributions presented in this study are included in the article/supplementary material, further inquiries can be directed to the corresponding author.

## Ethics statement

The studies involving human participants were reviewed and approved by Foreign Languages School, Huanghuai University, Zhumadian, China. The patients/participants provided their written informed consent to participate in this study.

## Author contributions

SZ wrote the whole manuscript from introduction to conclusion and approved the manuscript for submission.

## Appendix

### Entrepreneurial passion

ENP1. It is exciting to figure out new ways to solve unmet market needs that can be commercialized.

ENP2. Searching for new ideas for products/services to offer is enjoyable to us.

ENP3. We always motivated to figure out how to make existing products/services better.

ENP4. Scanning the environment for new opportunities really excites us.

ENP5. Inventing new solution to problems is an important part of business innovation.

### Entrepreneurial learning

ENL1. Frequently come up with creative ideas that challenge conventional ideas.

ENL2. Frequently experiment with radical new ideas (or ways of doing things).

ENL3. Always test the new ideas within the unknown field.

ENL4. Collected novel information and ideas that went beyond our current market and technological experiences.

ENL5. Improve existing technologies to strengthen the ability of R&D.

### Curiosity

CUR1. Everywhere I go, I am out looking for new things or experiences.

CUR2. View challenging situations as an opportunity to grow and learn.

CUR3. Like to do things that are a little frightening.

CUR4. Prefer jobs that are excitingly unpredictable.

CUR5. Frequently seek out opportunities to challenge myself and grow as a person.

CUR6. I am the kind of person who embraces unfamiliar people, events, and places.

### Business model innovation

BMI1. The business model offers new combinations of products, services, and information.

BMI2. Incentives offered to participants in transactions are novel.

BMI3. The business model gives access to an unprecedented variety and number of participants.

BMI4. The business model links participants to transactions in novel ways.

BMI5. The business model adopts a new way of trading.

BMI6. The business model creates a new profitable way.

BMI7. The business model creates a new profit point.

BMI8. The business model introduces new ideas, methods and commodities.
